# Inaccuracy of Language

**Published:** 1902-06-14

**Authors:** 


### INACCURACY OF LANGUAGE.

An article on "Details in Abdominal Section" which appears in the pages of an esteemed contemporary is full of interesting information and at the same time is so full of those curious forms of speech and inaccuracies of language into which medical writers are apt to fall, that, if for no other reason, it is worthy of attention. We are told that "overnight a compress made of boiled lint in perchloride of mercury is applied to the abdominal wall, which is well washed with soap and water, scraped with a razor." Of course we know what the writer means, but why cannot he say it in reasonably exact language. Further on we are told that "in a good case, the ordinary farinaceous diet, with fish and chicken, is given on the third day after the bowels have acted." Now does the writer mean the third day after the operation, or does he really mean what he says, namely, "the third day after the bowels have acted"?

"A gentle douche of boracic, formalin, or chinosol is often given to every hysterectomy, but this is a matter of judgment." What is meant by "often" and "every"? and how can it be a matter of judgment if it is given "to every hysterectomy"?

At the commencement of one paragraph we are told that "the pain in gynæcological operations is often intense in the small of the back," while at the end of the same paragraph and in reference to the same pain it is said that "it is not a common or urgent symptom as a rule," which seems somewhat a contradiction; while the explanation of the pain seems to display still greater laxity of thought, for it is said that this pain is "probably a referred pain due to the cervical nerves and irritation after incision into the dorsal." That the subject should agree with a verb is a grammatical detail to which it is perhaps unfair to allude in these hurried days and the construction of the following sentence only shows, perhaps, that the writer's mind was full of other and more important affairs: "For persistent vomiting, nutrient enemata with no fluids by mouth often answers; large draughts of soda-water 0 ss. followed by 0 ss. if the fluid is returned often brings away green acrid fluid with decomposing mucus, after which the vomiting may cease."

When the writer says that "champagne, iced or not, iodine water, oxylate of cerium and many others are used," we are almost as much puzzled to discover what the word "others" refers to as to find out why "oxalate" should be spelled with a "y." Although we are often told that the writing of prescriptions is a lost art, we ought at least so to write that our prescriptions can be dispensed. It may for example be hypercritical to suggest that there is something grammatically incorrect in the term "Sodæ Alba," but what, after all, is Sodæ Alba? The name does not appear either in the British Pharmacopœia or in the Pharmacopœia of the hospital from which this interesting paper comes. Has there been a slip of the pen, and is it, perchance, the preparation known as white magnesia that the writer is aiming at? As another example of inexact language we quote the following:—"Cellulitis and thrombosis round the stump, with adhesions, may cause intestinal obstruction and a localised abscess to pass unnoticed while the patient is being drenched with iced champagne to stop the vomiting." Floating in the writer's mind is evidently an idea which is perfectly correct, namely, that cellulitis round the stump, with adhesions, may cause intestinal obstruction and localised abscess, all of which may pass unnoticed, etc. But what he *says* is that the cellulitis may cause these things to pass unnoticed, which is quite a different thing. Students are taught to observe facts. If they were equally well taught to record what they observe in grammatical language, so that their meaning should be clear and indisputable, they would gain greatly in exactitude of thought, as well as standing higher than they do in the esteem of their examiners, which we suppose would by some be regarded as a matter of still greater importance.

